# Schmallenberg Virus Circulation in High Mountain Ecosystem, Spain

**DOI:** 10.3201/eid2006.130961

**Published:** 2014-06

**Authors:** Xavier Fernández-Aguilar, Joan Pujols, Roser Velarde, Rosa Rosell, Jorge R. López-Olvera, Ignasi Marco, Marti Pumarola, Joaquim Segalés, Santiago Lavín, Oscar Cabezón

**Affiliations:** Centre de Recerca en Sanitat Animal, Barcelona, Spain (X. Fernández-Aguilar, J. Pujols, R. Rosell, J. Segalés, O. Cabezón);; Universitat Autònoma de Barcelona, Barcelona (X. Fernández-Aguilar, R. Velarde, J.R. López-Olvera, I. Marco, M. Pumarola, J. Segalés, S. Lavín, O. Cabezón);; Institut de Recerca i Tecnologia Agroalimentàries, Barcelona (J. Pujols);; Generalitat de Catalunya Departament d’Agricultura, Barcelona (R. Rosell)

**Keywords:** Schmallenberg virus, viruses, Spain, mountains, circulation, ecosystem, vector-borne infections

**To the Editor:** Schmallenberg virus (SBV) is an emerging vector-borne virus mainly associated with *Culicoides* spp. midges (*1*,*2*). Factors affecting the density and distribution of vectors may help determine the prevalence of SBV infection in particular areas. Altitude could be one limiting factor for virus transmission; however, little information is available regarding SBV in high-altitude regions.

During December 29, 2012–February 21, 2013, morphologic anomalies were identified in 4 stillborn calves from different farms in northeastern Spain, and infection with SBV was suspected. The cases were clustered in the Ripollès and Garrotxa regions of Catalonia and appeared in beef cattle herds that spent the grazing season (May–November) in the alpine meadows (>2,000 m altitude) of the National Game Reserve of Freser-Setcases in the Eastern Pyrenees Mountains. The calves had severe arthrogryposis, ankylosis of several joints, abnormal curvature of the vertebral column, and severe muscle atrophy. Malformations of the central nervous system included bilateral hydrocephalus, cerebellar hypoplasia, and micromyelia, characterized by the presence of few neurons in the ventral horns and moderate to severe bilateral reduction of white matter in the ventral and lateral funiculi. 

SBV infection was confirmed by real-time reverse transcription qualitative PCR (RT-qPCR) (*1*,*3*) or serologic testing in 3 of the 4 calves and all 4 of the mothers (Table). Serum samples were tested by using a commercial indirect ELISA (ID.vet; Innovative Diagnostics, Montpellier, France) and a virus neutralization test using the BH80/11–4 isolate (provided by the Friedrich-Loeffler-Institut, Isle of Riems, Germany) (*4*). Consistent results were obtained from both of these techniques, and the proportions of calves positive by ELISA and RT-qPCR were similar to those found in previous studies (*5*).

**Table:** Results of serologic and molecular analyses of SBV in sympatric wild and domestic ruminants, Eastern Pyrenees, Spain, 2010–2013*

Ruminants and SBV cases	No. animals	SBV serologic testing results											RT–qPCR
		2010		2011		2012					2013		
						Mar–Aug		Sep–Dec†					
		Ratio		Ratio		Ratio		Ratio	Prev, % (95% CI)		Ratio	Prev, % (95% CI)	
Wild													
Chamois‡	260	0/45		0/89		0/21		5/81	6.2 (0.9–11.4)§		3/24	12.5 (0–25.7)§	–
Roe deer¶	20	–		0/6		0/9		–	–		4/5	80.0 (44.9–100)	–
Mouflon#	75	–		0/29		0/21		–	–		0/25	–	–
Fetuses													
Chamois	7	–		–		–		–	–		0/7	–	–
Mouflon	1	–		–		–		–	–		0/1	–	–
Roe deer	1	–		–		–		–	–		0/1	–	–
Domestic**													
Cattle	130	–		0/9		–		26/30	86.7 (74.5–98.8)		79/91	86.8 (79.9–93.8)	–
Sheep	60	–		0/30		–		14/30	46.7 (28.8–65.5)		–	–	–
Goat	13	–		0/4		–		2/9	22.2 (0–49.4)		–	–	–
SBV cases													
Stillborn calves	4	–		–		–		0/1	–		2/3	–	1/4††
Mothers of calves	4	–		–		–		–	–		4/4	–	–

*SBV, Schmallenberg virus; ratio, no. positive/no. tested; prev, prevalence; RT-qPCR, real-time qualitative reverse transcription PCR; –, no data or not applicable. †First evidence of SBV circulation in the study area was a seropositive chamois on September 3, 2012. ‡Two sampling periods, March–May and August–December. Only March–May in 2013. §Differences not statistically significant. ¶Sampling period April–May. #Sampling period April–June. **Two sampling periods, October–November 2011 and November 2012–April 2013. ††Brain, thymus, and abomasum fluid samples positive; liver and kidney samples negative.

The neurologic and musculoskeletal lesions found in the calves indicated that fetal infection probably occurred at 5–6 months’ gestation (*6*). Gestation started in mid-April to mid-May; therefore, maternal infection most probably occurred in late summer 2012 (September–October), when cows were grazing in the alpine meadows.

We then performed a serologic study in domestic and sympatric wild ruminants from the National Game Reserve of Freser-Setcases, which comprises 20,200 ha of alpine and subalpine ecosystems. We analyzed serum samples from 355 wild ruminants hunted during August 2010–May 2013; species sampled included Pyrenean chamois (*Rupicapra pyrenaica*), European mouflon (*Ovis aries musimon*), and roe deer (*Capreolus capreolus*). We also analyzed samples from fetuses of these species obtained in April 2013 (Table), as well as animals from 8 cow herds and 4 sheep–goat mixed herds; a mean of 14 samples were collected per herd during 2 sampling periods (Table). Two of the mixed sheep–goat herds were sampled during both sampling periods. All serum samples underwent ELISA testing; positive results were confirmed by virus neutralization (*4*).

Domestic ruminants sampled during October–November 2011 were seronegative, whereas all farms sampled during November 2012–April 2013 had infected animals (Table). High mean seroprevalence was found in cow herds; 105 (86.8% [95% CI 80.7%–92.8%]) of 121 herds tested were infected. Seroprevalance was lower but still high for mixed sheep–goat herds; 16 (41% [95% CI 25.6%–56.5%]) of 39 herds were infected. The earliest evidence of SBV in the study area came from a seropositive Pyrenean chamois hunted on September 3, 2012; this date coincides with the estimated months when cows that delivered stillborn calves were infected. For wild ungulates tested from September 2012 onwards, overall SBV seroprevalence was statistically higher (χ^2^ 33.47, 2 d.f., p<0.0001) in roe deer (4/5, 80% [95% CI 44.9%–100%]) than in Pyrenean chamois (8/105, 7.6% [95% CI 2.5%–12.7%]) and mouflon (0/23). Differences in seroprevalence for summer through autumn 2012 compared with spring 2013 in Pyrenean chamois were not significant (Table).

Roe deer seroprevalence was similar to the 88.9% reported in Belgium in December 2011, which contrasted with the lower seroprevalence observed in red deer, 54.6%, for the same month in the same study (*7*). Differences in seroprevalence between wild host species might be related to differences in exposure to SBV vectors depending on habitat selection, vector feeding habits, or host-specific factors; altitude might be an additional factor affecting exposure (*8*). Thus, the lower altitude habitat selection of roe deer and the housing of domestic ruminants in valley areas could explain the higher seroprevalence observed in these species compared with that in Pyrenean chamois and mouflon.

All fetuses of wild ruminants had negative serologic test results for SBV, and no gross lesions indicating infection were observed (Table). However, the potential reproductive disorders that SBV infection can cause in these species are unknown. 

Our findings support the hypothesis that SBV can circulate in alpine meadows at >2,000 m altitude and confirm the appearance of SBV in late summer and autumn 2012 in the high mountain ecosystem of the Eastern Pyrenees in Spain. A variety of domestic and wild ruminants showed susceptibility to SBV infection, but differences in seroprevalence suggest different roles for sympatric ruminants in SBV epidemiology. The role of vector species in the transmission of SBV in alpine ecosystems should be analyzed.
