# Correction: Vol. 20, No. 6

**DOI:** 10.3201/eid2011.C32011

**Published:** 2014-11

**Keywords:** errata, erratum, correction

Animal testing data were described incorrectly in the article Schmallenberg Virus Circulation in High Mountain Ecosystem, Spain (X. Fernández-Aguilar et al.). Testing showed Schmallenberg virus in 105 (86.8% [95% CI 80.7%–92.8%]) of 121 animals from cow herds and 16 (41% [95% CI 25.6%–56.5%]) of 39 animals from mixed sheep–goat herds. The article has been corrected online (http://wwwnc.cdc.gov/eid/article/20/6/13-0961.htm).

